# LncRNA-PVT1 indicates a poor prognosis and promotes angiogenesis via activating the HNF1B/EMT axis in glioma

**DOI:** 10.7150/jca.60257

**Published:** 2021-07-25

**Authors:** Yongyan Bi, Jie Ji, Youxin Zhou

**Affiliations:** 1 Department of Neurosurgery & Brain and Nerve Research Laboratory, The First Affiliated Hospital of Soochow University, Suzhou, Jiangsu, China; 2 Department of Neurosurgery, Minhang Hospital, Fudan University, Minhang, Shanghai, China; 3 Department of Rehabilitation Medicine, Minhang Hospital, Fudan University, Minhang, Shanghai, China

**Keywords:** lncRNA-PVT1, HNF1B, miR-1207-3p, glioma, EMT

## Abstract

Recent studies identified that long non-coding RNAs (lncRNAs) exhibited critical roles in tumor migration and invasion. However, the roles of lncRNAs in glioma remain unclear. The aim of this study was to uncover the underlying mechanisms of glioma progression and provide potential therapeutic targets for its treatment in clinic. Our microarray study showed that lncRNA-PVT1 was significantly upregulated in glioma tissues and played an important role in cell proliferation, migration, invasion and angiogenesis. Our data showed that the expression of lncRNA-PVT1 was increased obviously and associated with advanced tumor stage, metastasis, invasion ability, and poor prognosis in glioma patients. Up-regulation of lncRNA-PVT1 was observed to promote glioma cells proliferation, and invasion abilities in vitro as well as tumor growth in vivo by regulating miR-1207-3p expression. Online software (TargetScan, miRDB and miR TarBase) were used to predict the regulating mechanisms of lncRNA-PVT1, miR-1207-3p and HNF1B, which were validated by dual-luciferase reporter gene system. In vivo tumor-bearing mice models were established to validate the cellular results. Therefore, we suggested that lncRNA-PVT1/miR-1207-3p/HNF1B axis might play critical roles in glioma progression, indicating that lncRNA-PVT1/miR-1207-3p/HNF1B signaling axis may serve as novel molecular targets for glioma prevention and treatment.

## Introduction

Human glioma that is derived from the neural ectoderm is the most common type of intracranial neoplasm, accounting for more than 50% 1. Although therapeutic techniques, such as surgery, radiotherapy, and chemotherapy for glioma have achieved great strides, the median survival of glioma patients is only 9-12 months 2-3. The poor prognosis and high recurrence rate of glioma is largely due to the abundant blood flow, rapid growth, susceptibility of invasion to surrounding normal brain tissues and resistance to radiotherapy and chemotherapy 4. The tumorigenesis of glioma is biologically complex, involving a series of pathophysiological changes, and expression changes of many genes and signaling pathways 5. Thus far, there is no definite conclusion about the underlying molecular mechanisms that mediate the tumorigenesis of brain glioma. Therefore, it is necessary to uncover the pathogenesis of glioma and develop effective diagnostic and therapeutic targets.

Long non-coding RNAs (lncRNAs) belong to ncRNA with a transcription length of more than 200 nt (nucleotides) and lack of protein coding functions 6. Recently, accumulated evidence showed that lncRNAs play crucial roles in cellular functions, such as cell proliferation, apoptosis, differentiation, metastasis, drug resistance 7-8. Plasmacytoma variant translocation 1 (lnc-PVT1) is a lncRNA that has been found to serve an oncogenic role in a variety of malignant tumors 9. Recent evidence further indicates that lncRNA-PVT1 exhibits aberrant expression in nonsmall-cell lung carcinoma 10, cervical carcinoma 11, colorectal carcinoma 12, and gastric carcinoma 13. Located at 8q24, lncRNA-PVT1 has been identified as a candidate oncogene and highly expressed in multiple human neoplasms, which exerts regulatory functions in biological processes, such as proliferation, apoptosis, mobility, and invasion 14. Moreover, lncRNA-PVT1 expression is significantly linked to patient survival in those with breast carcinoma, lncRNA-PVT1 has been shown to directly bind and stabilize the KLF5 proteins in breast cancer 15. EZH2 forms a molecular complex with lncRNA-PVT1 to function as a repressive driver of p15 and p16 in gastric cancer 16. LncRNA-PVT1 is also transcriptionally activated by FOXM1 in gastric cancer 17. Furthermore, lncRNA-PVT1 induces radioresistance by influencing cell apoptosis and DNA repair in NPC 18. Furthermore, the specific role of lncRNA lncRNA-PVT1 in the pathogenesis of glioma has become a viewpoint, as there were quite a few studies on the roles of lncRNA-PVT1 in glioma 19-20. However, the underlying lncRNA-miRNA-mRNA regulatory networks involved with lncRNA-PVT1 in glioma are currently lacking.

MicroRNA has been frequently implicated in various cellular processes, including cell proliferation and apoptosis 21-23. Emerging evidences revealed that miR-30 family was related with tumor develepment. For example, Zhao *et al.* showed that miR-30-5p functioned as a tumor suppressor by regulating Wnt/β-catenin-BCL9 axis 24. Han *et al.* revealed that there is a feedback loop between miR-30a/c-5p and DNMT1 in the area of ovarian cancer cells resistance to cisplatin 25. Gao *et al.* found that miR-30c-5p reduced invasion and EMT processes of gastric cancer via targeting MTA1 26. Some previous researches have indicated that miR-1207-3p was significantly upregulated, which might be potential cancer oncogene in different types of cancers, such as prostate cancer 27, gastric cancer 28, pancreatic cancer 29, and colorectal cancer 30. Besides, miR-1207-3p has been found to exhibit a low expression and inhibit cell proliferation, suppress tumor growth, and block angiogenesis in medulloblastoma 31. Nevertheless, the regulatory function of lncRNA-PVT1/miR-1207-3p axis in glioma cells needs to be further explored.

Herein, the purpose of the present study was to investigate the explicit role of lncRNA-PVT1 in the pathogenesis of glioma as well as the potential mechanism of lncRNA-PVT1/ miR-1207-3p/HNF1B axis in glioma. In the present study, we found that lncRNA-PVT1 was upregulated in glioma, and that it predicted poor survival in patients. We delved into the lncRNA-miRNA-mRNA networks by co-expressing lncRNA and mRNA with altered miRNA. Bioinformatics analysis was used to predict possible pathways in which these networks might be involved.

## Materials and Methods

### Tissue samples collection

In our current study, glioma patient tissues and matched adjacent-tumor controls were obtained from the Department of Neurosurgery at Minhang Hospital of Fudan University, between May 2015 and December 2019. The collected tissues were immediately snap-frozen in liquid nitrogen and stored at -80°C before using. All the patients read and signed the informed consent forms and this study was approved by the Research Ethic Committee of the Fudan of University. Before surgery, none of the patients had received chemotherapy or radiation therapy.

### Cell culture and transfection

Human glioma cell lines U87, SHG-44, U251, H4, human umbilical vein endothelia cells (HUVEC), human embryonic kidney 293T (HEK293T) cell line and normal cells (HEB) were purchased from the Cell Resource Center of Shanghai Institutes for Biological Sciences (Shanghai, China). Human microglia (HM, ATCC® CRL‐3304^TM^) was purchased from the American Type Culture Collection (Manassas, VA). Glioma cells were cultivated in DMEM containing 10% fetal bovine serum (FBS; Sigma-Aldrich, St.Louis, MO, USA) in an incubator with constant temperature containing 5% CO_2_ at 37°C. Glioma cells were transduced with lentiviral vector suppressing lncRNA-PVT1 (sh-lncRNA-PVT1), miR-1207-3p mimics, miR-1207-3p inhibitors and corresponding negative controls. All synthesis (sh-lncRNA-PVT1, miR-1207-3p mimics, miR-1207-3p inhibitors) are done by Shanghai IBSBIO. Cells were transfected with shRNA or miRNA via Lipofectamine 3000 reagents (Invitrogen, USA).

### RNA extraction and quantitative real-time PCR

Total RNA was extracted from these included Glioma cells using TRIzol reagent based on the manufacturer's instructions 32. The cDNA was synthesized using Reverse Transcription Kit (Toyobo, Japan). qPCR assay was conducted using SYBR Green Real-time PCR Mix (Toyobo, Japan) and determined in triplicate. The primer sequence of lncRNA-PVT1: 5'-GGGGAATAACGCTGGTGGAA-3', 5'-CCCATGGACATCCAAGCTGT-3'. Data were normalized to GAPDH expression. The relative expression of target genes were analyzed by the 2-ΔΔCt method.The PCR program was set as follows: 50˚C for 2 min firstly, followed by 95˚C for 10 min, and 38 cycles of 95˚C for 15 s and 60˚C for 1 min. U6 and GAPDH were served as internal reference.

### Microarray analysis

Total RNA was extracted from 3 paired glioma tissues, Tissue samples preparation and microarray hybridization were performed according to the instructions. The raw data and array images were extracted with Agilent Feature Extraction (version11, Agilent, USA) software. Subsequent data processing was done using the GeneSpring GX v12.0 (Agilent). The microarray was provided by KangChen (Shanghai, China). The threshold cutoff was a fold change ≥2.0 and a P value ≤0.05 for downregulated and upregulated lncRNAs.

### Western blot assay

Total proteins from cells were extracted using RIPA lysis buffer (Thermo Fisher, USA) and protein concentrations were measured with BCA protein assay (Pierce, IL, USA). Totally 45 μg of each protein was separated through 15% SDS-PAGE gels and transferred onto PVDF membranes according to the instructions (Millipore, USA). After blocking with 5% skimmed milk, the membranes were treated with primary antibodies at 4℃ overnight. Then the corresponding secondary antibody was used for 1h at room temperature. Protein bands were visualized by ECL (Thermo Fisher, USA). Soluble proteins were analyzed by immunoblotting with anti-HNF1B (07-2192; Sigma-Aldrich), anti-foxm1 (SAB3300002; Thermo Scientific), anti-Vimentin (ab8069; Abcam), anti-N-Cadherin (ab18203; Abcam), anti-p-ERK (mAb #4370), anti-ERK (mAb #4695), anti-p65 (mAb #8242), anti-ZEB1 (mAb #3396; Cell signaling Technology) and anti-GAPDH (G8795; Sigma).

### Immunohistochemistry staining and scoring

The experimental methods are based on previous study. Specifically, staining intensity was scored manually by two independent experienced pathologists. For evaluation of IHC staining, a semiquantitative scoring criterion was used, in which both staining intensity and positive areas were recorded. Obtained as the intensity of HNF1B positive staining (negative =0, weak =1, moderate =2, or strong =3 scores) and the proportion of immunopositive cells of interest (<10% =1, 10%-50% =2, >50% and <80% =3, >80% =4 scores), was calculated. In the case of heterogeneous staining, we determined the percentage of different staining intensities individually in each area and calculated the total sum (3). The following primary antibodies were used: anti-HNF1B (1:200 dilutions, Cell Signaling, Boston, USA).

### Cell proliferation assay

The cell proliferation viability was assessed by MTT assay (IBSBIO, China). Cells were seeded on 96-well plates in triplicates at the density of 1000-2000 cells/100 ml. Thereafter, 20 μl of MTT solution was added and incubated at 37°C for 5 h. The reaction liquid was abandoned, then 200 µl of DMSO was added. Finally, the optical density 470 (OD 470) absorbance values were obtained in triplicate and replicated 6 times.

### Cell cytoplasm/nucleus fraction isolation

To prepare cytoplasmic and nuclear extracts from glioma cells, NE-PER Nuclear and Cytoplasmic Extraction Reagents (Thermo Scientific, Waltham, MA, USA) was used. RT-qPCR was employed to analyze the levels of nuclear control transcript, cytoplasmic control transcript and lncRNA-PVT1.

### Transwell assay

Cell invasion ability was carried out by Transwell invasion assay. Briefly, transfected cells were seeded into 24-well upper chamber (Corning, CA USA) pre-coated with Matrigel (Sigma, MO, USA). The lower chamber was filled with DMEM medium containing 10% FBS. After culturing for 24 h, the invade cells were stained with 0.1% crystal violet (Sigma) for 10 min. The number of invaded cells was counted under a light microscope (Nikon, Japan).

### Luciferase reporter assay

lncRNA-PVT1 and HNF1B 3'UTR Wt and mutant DNAs were amplified by PCR, then separately inserted into pmiR-GLO vector (Promega) to construct a Wt and Mut type of lncRNA-PVT1 and HNF1B plasmids. Cells were transfected with Wt or Mut reporter plasmid and miR-1207-3p mimics by lipofectamine 3000 reagent (Invitrogen). After 48 h of transfection, the luciferase activity was measured by Dual-luciferase reporter assay system (Promega, CA, USA).

### Tumor xenograft implantation in nude mice

All BALB/c nude mice were purchased from Shanghai Laboratory Animal Center (Shanghai, China). Before the experiments, the mice were acclimatized to the new environment for one week. Mice were randomized into different groups with approximately equivalent numbers before tumor cell inoculation. To determine the effects of lncRNA-PVT1 on tumor formation in vivo, glioma cells stably transfected with sh-lncRNA-PVT1 or sh-NC (2.0×10^6^ cells/site) were injected subcutaneously into 4-6 weeks old female mice. The developing tumors were observed during the following 7 weeks, after which the mice were sacrificed. The weights of each tumor were also documented. Tumor volume was calculated as V = length × (width^2^)/2. The protocol was approved by the Institutional Animal Care and Use Committee of Fudan University.

### Gene Set Enrichment Analysis (GSEA)

GSEA (http://software.broadinstitute.org/gsea/msigdb/index.jsp) software programs were used to analyze The Cancer Genome Atlas (TCGA) dataset. To interpret the function of regulated genes, GSEA (version 2.2.0) was performed using the 50 cancer hallmark gene sets and a gene log2-fold change. To identify the pathways that are correlated with LncRNA-PVT1 expression in tumor samples, we performed a similar GSEA for each cancer type in TCGA dataset. In this analysis, GSEA was performed on the ranked list based on the Spearmen's correlation coefficient with LncRNA-PVT1 expression.

### Statistical analysis

In the present study, all of the statistical analysis was employed using SPSS 17.0 software package (SPSS Inc. Chicago, USA), GraphPad Prism 8.0 software (GraphPad, USA), and Microsoft office 2019 EXCEL (USA). *p* < 0.05 was considered statistically significant. Pearson χ^2^ or Fisher's exact test was performed to find out the associations between the indicators expression and clinicopathological parameters in our included glioma patients. Linear regression analysis was explored to analyze the correlation of lncRNA-PVT1 with other indicators. Student's *t*-test (two-tailed) and Wilcoxon or Welch's T-test were used to compare the significance differences of the paired and unpaired continuous variables between groups, presented as mean ± standard deviation (s.d.) or median (quartile).

## Results

### Microarray analysis demonstrates significant alterations in lncRNA expression patterns in human glioma

**Figure 1:**
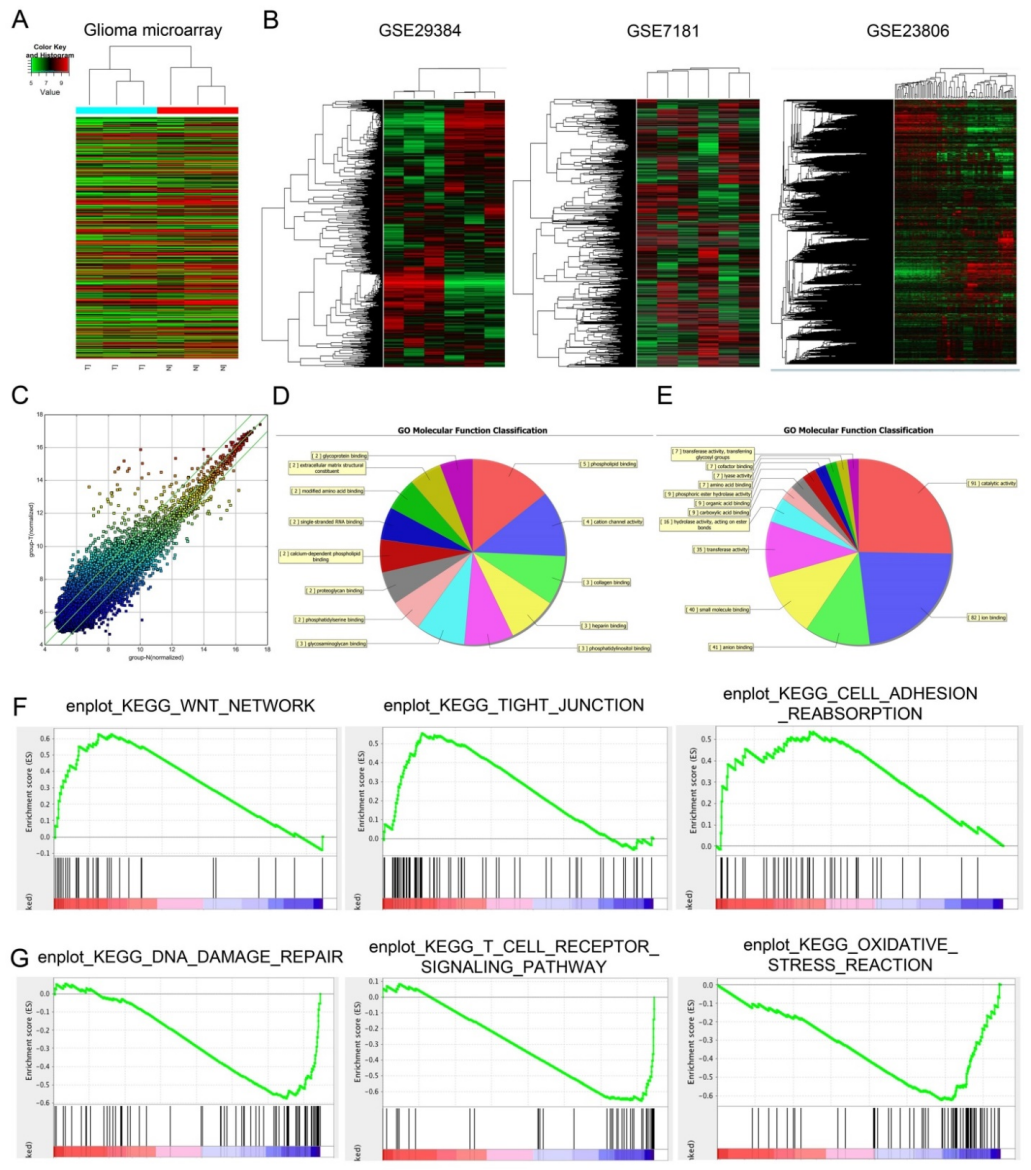
Dysregulated expression profiles of lncRNAs in glioma. (A) Aberrantly expression of lncRNAs was evaluated in 3 pairs of glioma and adjacent normal tissues though lncRNA expression microarray. (B) lncRNA-PVT1 expression in microarray datasets GSE29384, GSE7181 and GSE23806. (C) Scatter plot of gene expression in glioma compared with the control group (x-axis). Up-regulated lncRNAs are shown in red while down-regulated lncRNAs are shown in green. (D, E) GO biological process classification for differently expressed lncRNAs. The upper graph was related to the up-regulated lncRNAs (D) while the lower graph was related to the down-regulated lncRNAs (E). (F, G) GSEA revealed that proliferation and metastasis related biological functions were enriched in response to high lncRNA-PVT1 expression based on the microarray data set.

Differentially expressed lncRNAs (fold change ≥ 1.5 and *p* < 0.05) were observed in **Figure 1A**, there are 327 lncRNAs exhibited high expression levels and 559 lncRNAs exhibited low expression levels. Among the dysregulated lncRNA transcripts, the mostly upregulated lncRNAs were lncRNA-PVT1 and lnc-004831, of which lncRNA-PVT1 was the highest (log_2_FC = 4.32). In an attempt to further explore the function of lncRNA-PVT1 in a lncRNA-dependent manner, we first focused on 3 microarray data, GSE29384, GSE7181 and GSE23806 through searching GEO datasets **(Figure 1B)**. As shown in **Figure 1C**, 327 genes were upregulated and 559 genes were downregulated, Scatter plot of gene expression in glioma compared with the control group. Then, gene ontology analysis (GO) was used to explore the roles of these differentially expressed lncRNAs. Results showed the majority of lncRNAs were assessed to be related to cancer progression (molecular function, cellular components and biological processes) **(Figure 1D, E)**. Subsequently, GSEA results from microarray database showed that high lncRNA-PVT1 expression was associated with wnt pathway, cell adhesion and disease free survival of patients with glioma **(Figure 1F, G)**. Therefore, we suggested that lncRNA-PVT1 might play critical roles in glioma progression.

### High lncRNA-PVT1 expression in glioma cells and tissues

**Figure 2:**
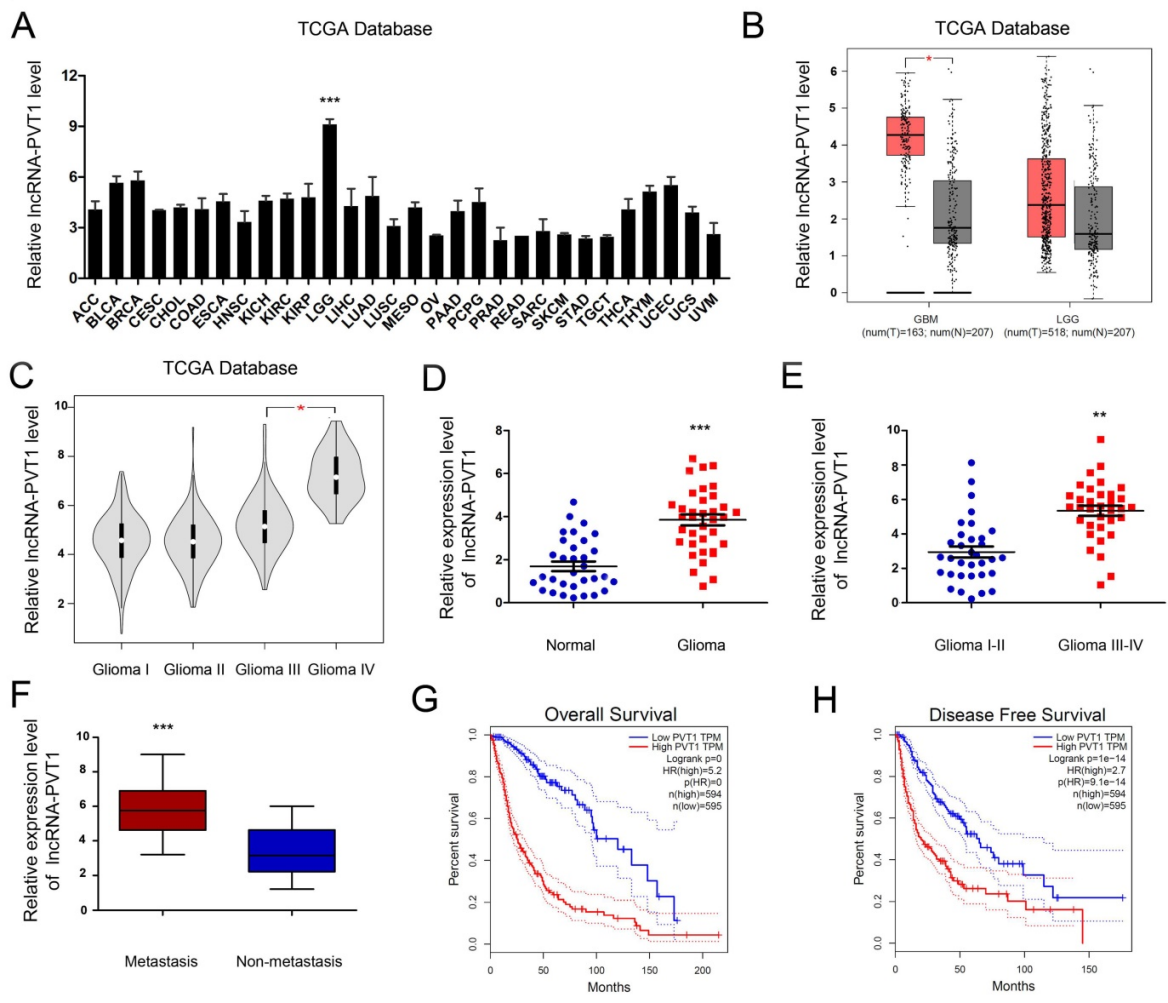
lncRNA-PVT1 was upregulated in glioma. (A, B) lncRNA-PVT1 expression in multiple solid tumors and normal tissues from TCGA Pan-Cancer dataset. (C) lncRNA-PVT1 expression in different TNM stage of glioma cases from TCGA dataset. (D) The lncRNA-PVT1 expression in glioma cases were determined by qRT-PCR assay. (E, F) High lncRNA-PVT1 expression was positively associated with advanced TNM stage and metastasis in patients with glioma. (G, H) The Kaplan-Meier curves introduced for survival analysis in glioma patients with high level and low level of lncRNA-PVT1. **p<0.01, ***p<0.001.

To enrich the dysregulated lncRNAs-mRNAs and their correlations, the tumor development data were collected from TCGA Pan-Cancer database. The results illustrated that the expression of lncRNA-PVT1 was pronouncedly increased in glioma tumor tissue compared to normal tissues. In most other solid tumors, such as breast invasive carcinoma, cervical squamous cell carcinoma and endocervical adenocarcinoma and cholangio carcinoma, lncRNA-PVT1 showed the opposite expression level **(Figure 2A, B)** (*p* < 0.001). Further, the expression level of lncRNA-PVT1 in glioma gradually increased with the increase of clinical stage, and the difference was significant (*p* < 0.05)** (Figure 2C)**.

To assess the roles lncRNA-PVT1 in glioma, qRT-PCR showed that lncRNA-PVT1 was significantly upregulated in glioma tissue compared to adjacent non-tumor tissues **(Figure 2D)**. High lncRNA-PVT1 expression was increased in glioma patients with advanced TNM stage and metastasis **(Figure 2E, F)**. Furthermore, we analyzed the relationship between lncRNA-PVT1 expression and clinicopathologic features. Results showed that high lncRNA-PVT1 expression was associated with advanced tumor stage, metastasis, OS and DFS **(Figure 2G, H)**. These data indicated that lncRNA-PVT1 might be involved in glioma metastasis.

**Figure 3:**
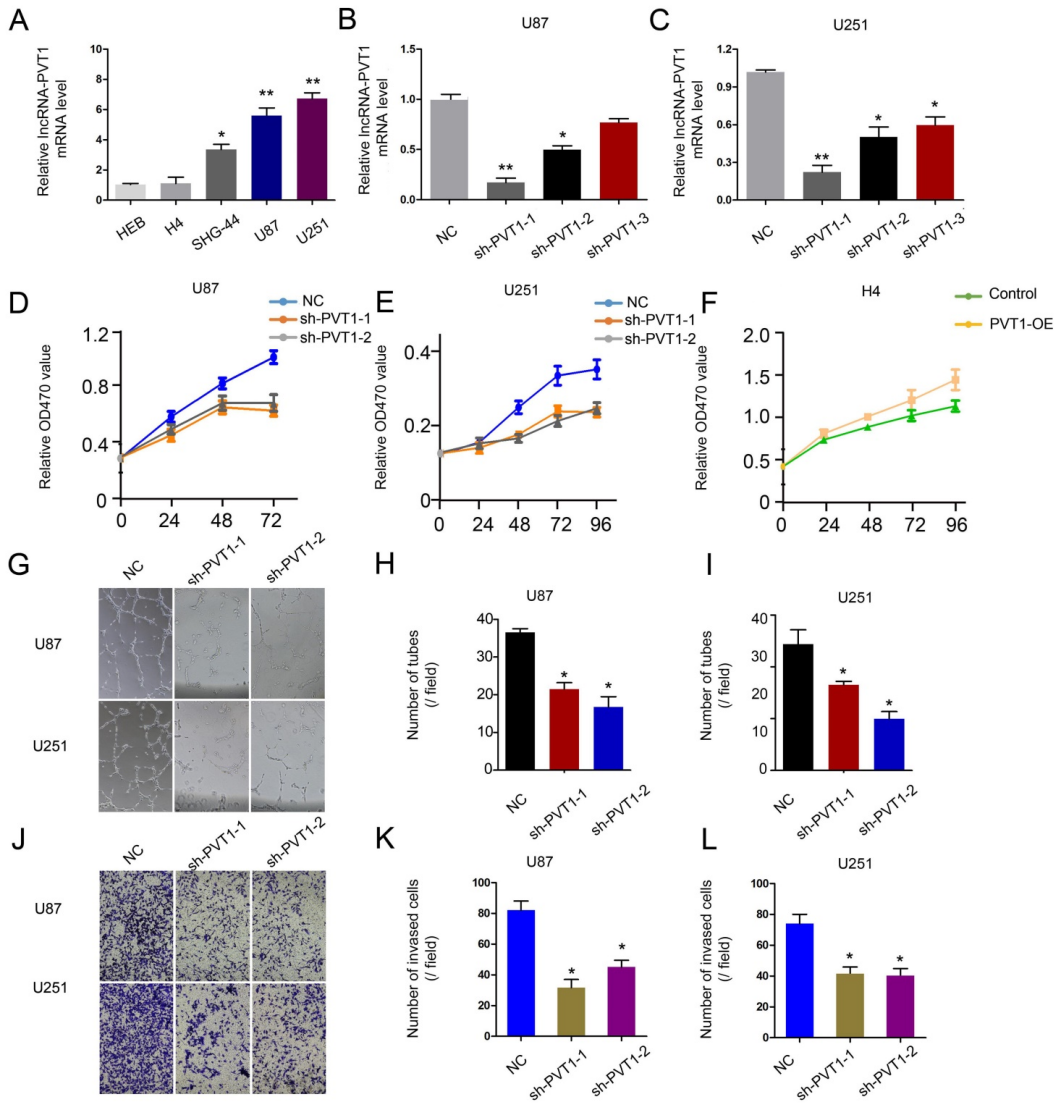
lncRNA-PVT1 promotes glioma the development of pro-metastatic phenotype in *vitro*. (A) lncRNA-PVT1 expression in glioma cell lines (H4, SHG-44, U87 and U251) and HEB was detected by qRT-PCR assay. (B, C) Knockdown of lncRNA-PVT1 by shRNA in U87 and U251 cells. (D-F) MTT assay was performed to assess the function of lncRNA-PVT1 in glioma cells proliferation. (G-I) The supernatants from sh-lncRNA-PVT1 cells exhibited a strong negative effect on tubule formation of HUVECs, compared to control supernatants in U87 and U251 cells. (J-L) Knockdown of lncRNA-PVT1 in U251 and U87 cells significantly inhibited cell invasion. We selected the 1# and 2# sh-plasmids with high interference efficiency for testing. *p<0.05.

To further determine the underlying mechanisms of lncRNA-PVT1 on glioma progression, loss-of-function assays were used. The expression of lncRNA-PVT1 was significantly reduced in U251 and U87 cells transfected with sh-lncRNA-PVT1 **(Figure 3A-C)**. MTT assays showed that lncRNA-PVT1 suppression reduced the proliferation ability of U251 and U87 cells** (Figure 3D-F)**. Human umbilical vein endothelia cells (2 × 10^4^ cells / well) were resuspended in the supernatant from sh-lncRNA-PVT1-U251 or sh-NC-U251 cells, and sh-lncRNA-PVT1-U87 or sh-NC-U87 cells. Then number of junctions and the total tubule length were chosen to assess the differences groups through Image *J* software. Next, the supernatants from sh-lncRNA-PVT1-U251 and sh-lncRNA-PVT1-U87 cells exhibited a strong negative effect on tubule formation of HUVECs, compared to control supernatants (*p* < 0.05) **(Figure 3G-I)**. Transwell assays showed a similar result as tubule formation assays, sh-lncRNA-PVT1 significantly repressed the cell invation of glioma cancer cells **(Figure 3J-L)**.

### Identification of lncRNA-PVT1 target genes

**Figure 4:**
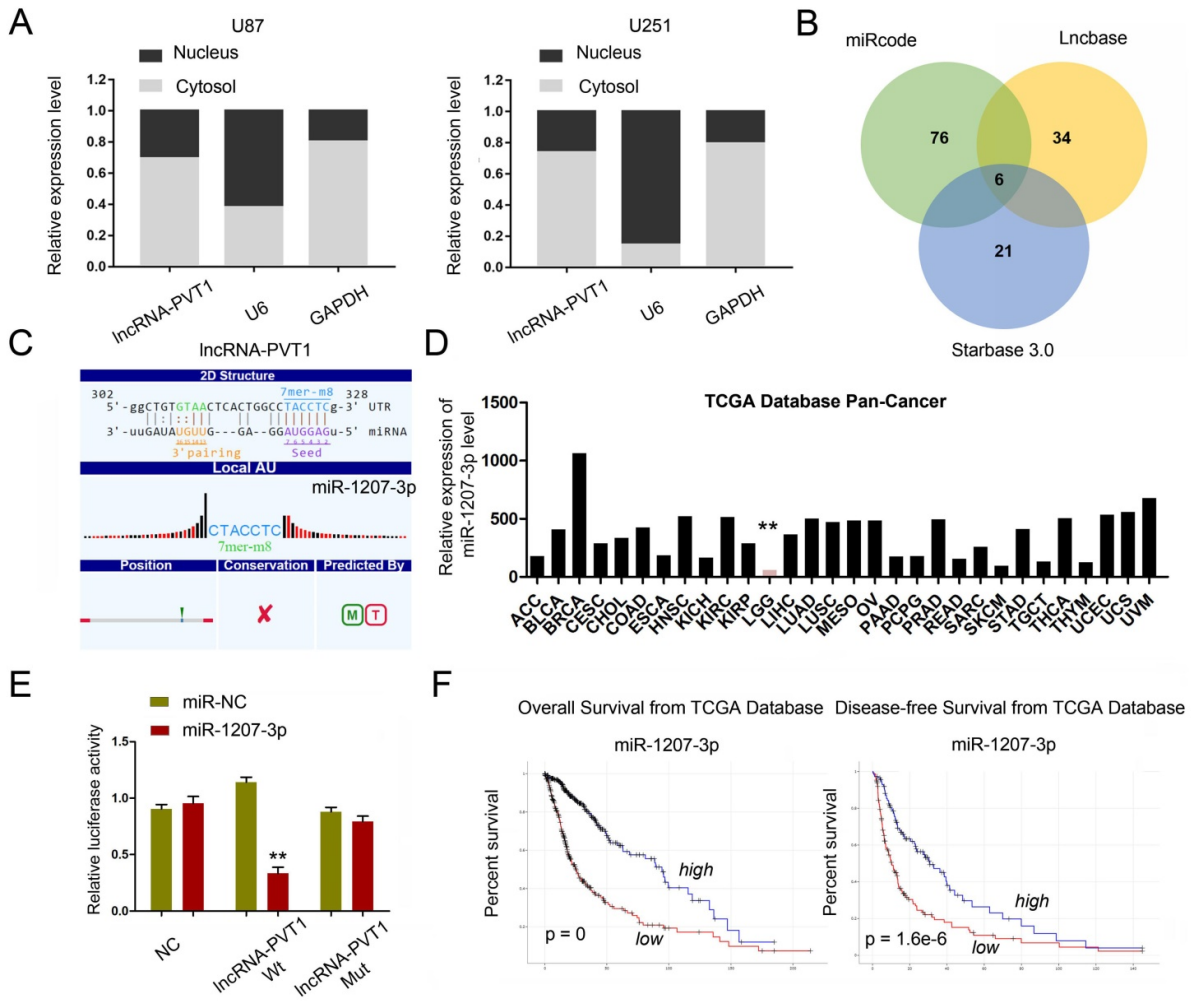
lncRNA-PVT1 acted as a sponge for miR-1207-3p. (A) lncRNA-PVT1 was located in the cytoplasm of U87 and U251 cells. (B) Venn diagrams showed the results of potential targets of lncRNA-PVT1 (LncBase Predicted, Starbase 3.0 and miRcode databases). (C) Bioinformatics analysis shows that lncRNA-PVT1 contains one conserved target binding site of miR-1207-3p, the predicted complementary binding sites between lncRNA-PVT1 and miR-1207-3p. (D) miR-1207-3p expression in multiple solid tumors and normal tissues from TCGA Pan-Cancer dataset. (E) Dual-luciferase assay showed miR-1207-3p mimics reduced the luciferase activity of lncRNA-PVT1-Wt. (F) Low miR-1207-3p expression was associated with poor OS and DFS in patients with glioma. **p<0.001.

To further explore whether lncRNA-PVT1 serve as an endogenous RNAs (ceRNA) to exerts its function in glioma cells, we first examined lncRNA-PVT1 expression in the cytoplasmic and nuclear fractions of glioma cells. The results indicated that lncRNA-PVT1 was in the cytoplasm, which is the pre-condition of acting as a ceRNA **(Figure 4A)**. We found that miR-1207-3p ranked top among all potential targets by bioinformatics analysis (miRcode, Starbase, and LncBase Predicted), the secondary structure and possible binding sites was shown in **Figure 4B**. The results illustrated that the expression of miR-1207-3p was pronouncedly increased in TCGA Pan-Cancer database **(Figure 4D)**. The dual-luciferase reporter assay showed that the luciferase activity of HEK293 cells transfected with lncRNA-PVT1-Wt could be reduced by miR-1207-3p mimics **(Figure 4E)**. Kaplan-Meier analysis in the TCGA database with glioma revealed that high lncRNA-PVT1 expression level was significantly correlated with a reduction in OS and DFS **(Figure 4F)**, which is consistent with the important role of lncRNA-PVT1 in the pathogenesis and the prognosis of glioma in our study.

### lncRNA-PVT1 regulates the expression of HNF1B by competitive adsorption of miR-1207-3p

**Figure 5:**
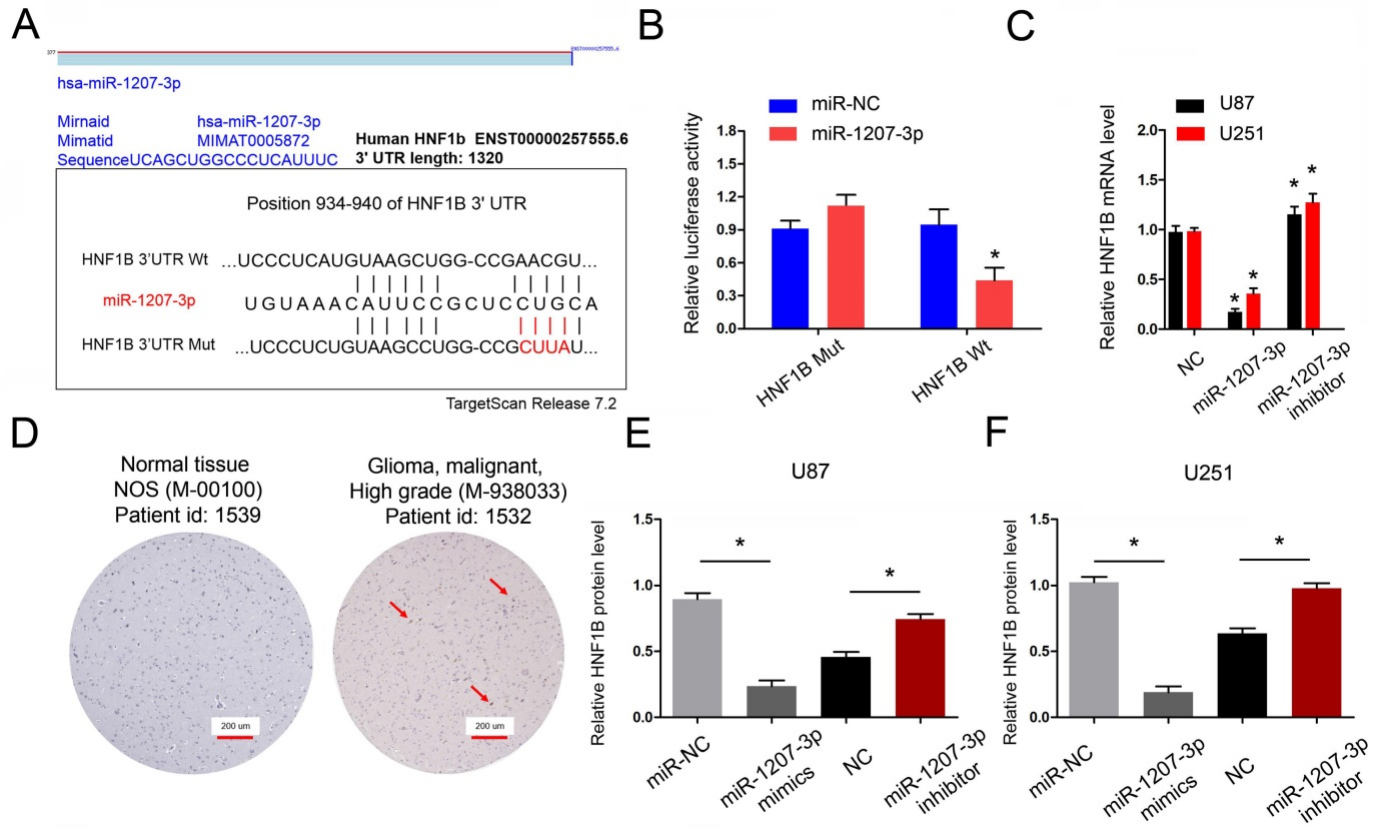
HNF1B is the direct target gene of miR-1207-3p and associated with poor prognosis of glioma. (A) The predicted complementary binding sites between miR-1207-3p and HNF1B. (B) Dual-luciferase assay showed miR-1207-3p mimics reduced the luciferase activity of HNF1B-Wt. (C) miR-1207-3p mimics reduced HNF1B expression in glioma cells, while miR-1207-3p inhibitors increased HNF1B mRNA expression. (D) IHC assays showed HNF1B expression was upregulated and associated advanced TNM stage in patients with glioma. (E, F) Western blot results showed overexpression of miR-489 significantly down-regulated HNF1B in U87 and U251 cells. *p<0.05.

Next, we explored the functions of miR-1207-3p in glioma. We further predicted the targeting sites between miR-1207-3p and HNF1B and found that miR-1207-3p shared complementary binding sites with HNF1B at 3'-UTR **(Figure 5A)**. To verify the predicted results, dual-luciferase reporter assay showed that miR-1207-3p mimics significantly reduced luciferase activities of HNF1B-Wt group **(Figure 5B)**. In addition, miR-1207-3p mimics significantly decreased HNF1B mRNA expression, while miR-1207-3p inhibitors increased HNF1B mRNA expression **(Figure 5C)**. IHC results showed that both HNF1B protein expression was upregulated in glioma tissues and positively associated with advanced TNM stage **(Figure 5D)**, which was further confirmed by TCGA database. Western blot results showed that overexpression of miR-1207-3p significantly down-regulated HNF1B compared with the control group, suggesting that HNF1B expression is inversely regulated by miR-1207-3p in glioma **(Figure 5E, F)**. Those data indicated that HNF1B and foxm1 might play critical roles in glioma progression.

### lncRNA-PVT1/miR-1207-3p/HNF1B axis promoted glioma progression

**Figure 6:**
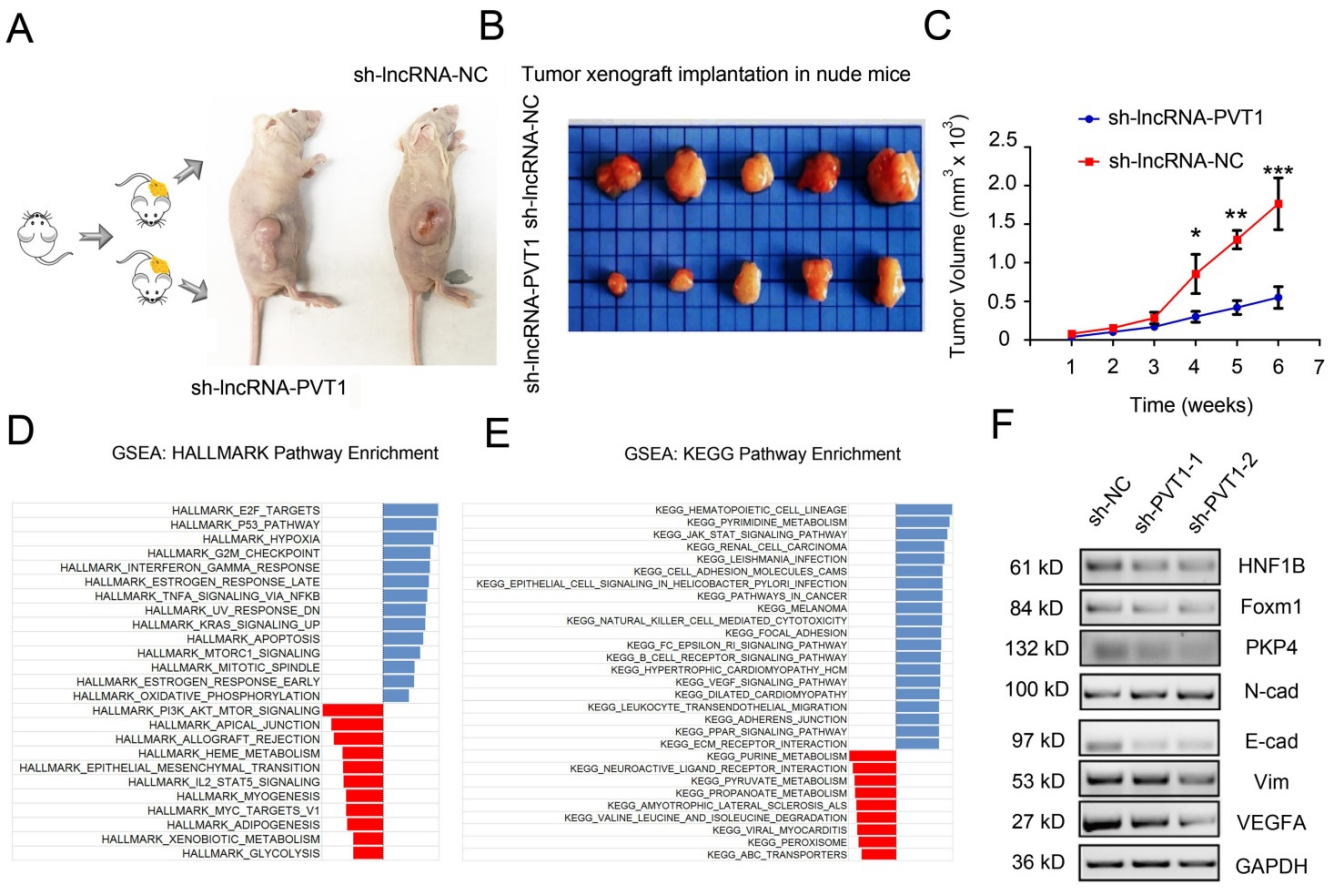
lncRNA-PVT1 inhibition reduced tumor growth in *vivo*, and lncRNA-PVT1/miR-1207-3p/HNF1B axis promoted glioma metastasis. (A) Representative images of tumors in xenograft mouse model. (B)The relative weights of xenograft tumors. (C) The growth curves of xenograft tumors. (D, E) HALLMARK and KEGG Pathway Enrichment in glioma from microarry datasets. The datasets were analyzed using the Hallmark gene signature collection. Hallmark_EMT was identified as the pathway with the top two-highest association. (F) The expressions of HNF1B, EMT-related makers and VEGF-related molecules were determined using western blot. Proteins were isolated from cells transfected as indicated. *p<0.05, **p< 0.01, ***p<0.001.

To determine whether the effects of lncRNA-PVT1 in glioma cells metastasis were dependent on miR-1207-3p/HNF1B axis, we further tested the roles of lncRNA-PVT1/miR-1207-3p/HNF1B axis in glioma progression. Furthermore, we examined the effects of lncRNA-PVT1 in xenograft mouse model. Results revealed that lncRNA-PVT1 inhibition also significantly reduced tumor growth in *vivo* **(Figure 6A-C)**. Overall, these results indicated that lncRNA-PVT1 silencing significantly reduced the proliferation and invasion ability of glioma cells in vitro and reduced tumor growth in *vivo*. EMT processes play critical roles in tumor metastasis. In the present study, western blot showed that lncRNA-PVT1 inhibition significantly reduced EMT related gene expression (Vimentin and N-cadherin) and MAPK pathway related gene expression (foxm1, VEGFA and PKP4) expression. And the effects of lncRNA-PVT1 inhibition on MAPK signaling pathway and EMT processes could be restored by miR-1207-3p shRNAs **(Figure 6F)**. Thus, our data suggested that lncRNA-PVT1 promoted the proliferation and metastasis of glioma by regulating miR-1207-3p/HNF1B/MAPK pathway **(Figure 6D-E)**.

## Discussion

At present, only a few functional mechanisms of lncRNAs have been well characterized, and some recent insights into lncRNAs identified in other diseases and cancers, such as molecules encoding tumor-related micropeptides, changes in the activities or conformation of proteins and the regulation of tumor microenvironment homeostasis, have not yet been clearly demonstrated in glioma pathogenesis 33. The majority of studies of glioma-associated lncRNAs are typically focused on ceRNA regulatory networks 34. Thus, there is an urgent need to elucidate the detailed mechanisms of the roles of lncRNAs in glioma.

Glioma displays complex etiology involving multiple risk factors, such as environmental exposure, inherited genetic alterations, and multiple epigenetic modifications based on global molecular biomarkers, including mRNAs and lncRNAs 35, 36. Although surgical resection is the best chance for a possible cure, the prognosis of glioma remains poor 37. Therefore, it is highly desirable to identify and characterize genes that play important roles in glioma pathogenesis. Among the critical molecules involved in the glioma progression, lncRNAs gradually become an attractive therapeutic target for cancers 38. For example, Yang *et al.* found that lncRNA-BANCR promoted non-small-cell lung cancer cells growth and metastasis via affecting EMT processes 39. Fu *et al.* found that lncRNA-HOTTIP modulated cancer stem cell properties through regulating HMGA1 in human gastric cancer 40. Jin *et al.* suggested that lncRNA-HOTTIP enhances osteogenic differentiation via interaction with WDR5 and up-regulation of β-catenin gene expression, therefore activating Wnt/β-catenin signalling pathway 41. Additionally, whether lncRNA expression in glioma is dysregulated and abnormal remains elusive, and the underlying mechanisms are still unknown.

LncRNA-PVT1 is reported to be involved in tumorigenesis and progression of many malignancies, including cervical cancer, nonsmall cell lung cancer, pancreatic cancer, and so forth 42. In the recent years, some emerging evidence has suggested that silencing PVT1 could inhibit malignant biological behaviors of glioma cells 43. For instance, Xue *et al.* unraveled that knockdown of PVT1 weakened the malignant behaviors of glioma cells via the inhibition of cell motility and invasiveness 44. Wang *et al.* also substantiated that downregulation of PVT1 expression resulted in decreased cell viability and mobility and increased apoptosis 45. In addition, lncRNAs can act as competing endogenous RNA sponges for miRNAs to regulate the degradation of miRNA targets, thereby influencing post-transcriptional regulation. In our study, we revealed that lncRNA-PVT1 was obviously increased in glioma tissues as well as in glioma cell lines. High lncRNA-PVT1 expression was positively associated with advanced TNM stage, metastasis, and poor prognosis in patients with glioma. In addition, loss-of-function assays demonstrated that lncRNA-PVT1 inhibition suppressed the proliferation and invasion in glioma cells in vitro and reduced tumor growth in *vivo*. Then the regulatory relationship between lncRNA-PVT1 and miR-1207-3p was validated through bioinformatics analysis, luciferase reporter assay as well as FISH assay. The present study further confirmed that transcriptional activation of lncRNA-PVT1 contributed to oncogenesis, whereas knockdown of lncRNA-PVT1 impaired oncogenic function of HNF1B in glioma cells. These data suggested that lncRNA-PVT1 could play critical roles in glioma tumorigenesis.

HNF1B could play critical functions in proliferation, metastasis, apoptosis, and linked to EMT pathway in several cancers 46-49. In our study, by bioinformatic prediction and experimental verification, we showed that HNF1B could act as a direct target of miR-1207-3p in glioma. Furthermore, we showed that the anti-metastatic roles of lncRNA-PVT1 inhibition on glioma cells invasion could be abolished by co-transfected with miR-1207-3p inhibitors (or pcDNA3.1_HNF1B plasmid). In addition, western blot revealed that lncRNA-PVT1 inhibition significantly reduced the expression of EMT pathway related genes. And the effects of lncRNA-PVT1 inhibition on EMT pathway could be restored by co-transfected with miR-1207-3p inhibitors. Therefore, these findings suggested that lncRNA-PVT1 promoted the proliferation and invasion in glioma via regulating miR-1207-3p-mediated HNF1B/EMT pathway.

**Figure 7:**
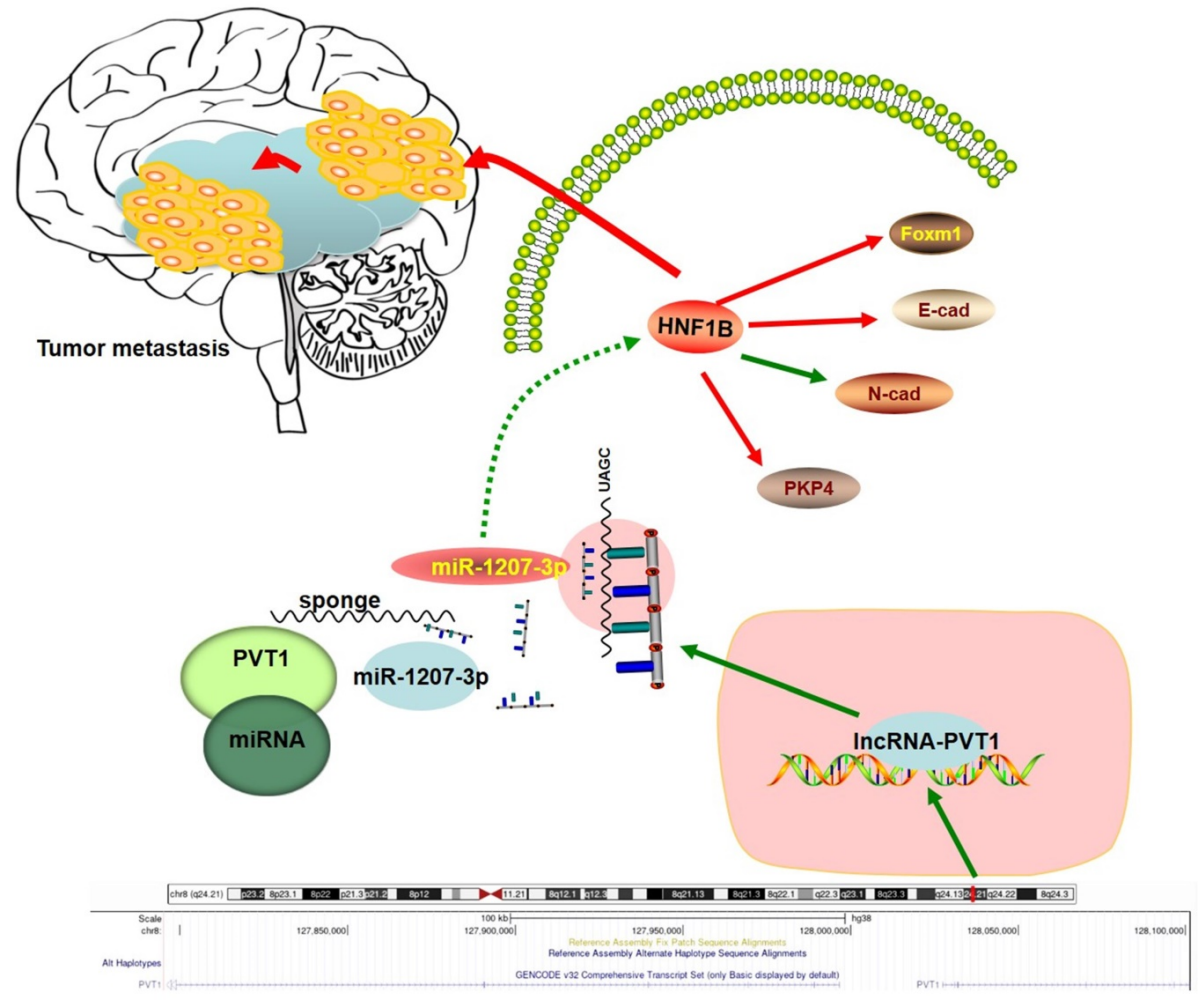
Schema diagram of lncRNA-PVT1-miR-1207-3p/HNF1B axis in human glioma.

In summary, our study identified the interaction between lncRNA-PVT1, miR-1207-3p and HNF1B in glioma **(Figure 7)**. In the present study, miR-1207-3p was predicted as the target of lncRNA-PVT1. Dual-luciferase reporter assay confirmed the correlation between lncRNA-PVT1 and miR-1207-3p. Furthermore, transwell invasion assay further revealed that lncRNA-PVT1 promoted glioma cells invasion by downregulating miR-1207-3p expression. Overall, we suggested that lncRNA-PVT1 could act as a sponge for miR-1207-3p in glioma. And the lncRNA-PVT1/miR-1207-3p/HNF1B/MAPK axis might provide a new potential therapeutic target for treatment of glioma.
